# A Fully-Immersive and Automated Virtual Reality System to Assess the Six Domains of Cognition: Protocol for a Feasibility Study

**DOI:** 10.3389/fnagi.2020.604670

**Published:** 2021-01-07

**Authors:** Jie En Lim, Wei Teen Wong, Tuan Ann Teh, Soon Huat Lim, John Carson Allen, Joanne Hui Min Quah, Rahul Malhotra, Ngiap Chuan Tan

**Affiliations:** ^1^Duke-NUS Medical School, Singapore, Singapore; ^2^SingHealth Polyclinics-Outram, SingHealth Polyclinics, Singapore, Singapore; ^3^Technology Development Centre, Institute of Technical Education College West, Singapore, Singapore; ^4^Centre for Quantitative Medicine, Duke-NUS Medical School, Singapore, Singapore; ^5^Head Office, SingHealth Polyclinics, Singapore, Singapore; ^6^SingHealth Duke-NUS Family Medicine Academic Clinical Programme, Duke-NUS Medical School, Singapore, Singapore; ^7^Centre for Ageing Research and Education, Duke-NUS Medical School, Singapore, Singapore; ^8^Health Services and Systems Research, Duke-NUS Medical School, Singapore, Singapore

**Keywords:** cognition, domain, assessment, virtual reality, dementia

## Abstract

**Introduction:** Dementia is increasing in prevalence in aging populations. Current questionnaire-based cognitive assessments may not comprehensively assess cognitive function and real-time task-performance. Virtual reality (VR) technology has been used in cognitive assessments but existing systems have limited scope in evaluating all cognitive domains. We have developed a novel, fully-immersive VR system (CAVIRE: Cognitive Assessment by VIrtual REality), which incorporates automated audio-visual instructions. An automated scoring matrix to assess the six cognitive domains—perceptual-motor function, executive function, complex attention, social cognition, learning and memory, and language—is embedded in the CAVIRE system.

**Aims:** The primary aim is to evaluate the feasibility of using the CAVIRE system to assess cognitive function of participants across different age groups from 35 to 84 years old. The secondary aims are to determine the CAVIRE performance-indices (completion time and scores) of the participants and to assess their acceptability toward the use of CAVIRE as a modality for cognitive assessment.

**Methods:** One hundred and seventy-five participants will be assessed by CAVIRE at a primary care clinic in Singapore. They will be first assessed using questionnaires: Montreal Cognitive Assessment (MoCA), Abbreviated Mental Test (AMT), Mini-Mental State Examination (MMSE), Basic Activities of Daily Living (BADLs), Instrumental Activities of Daily Living (IADLs). Those aged 65–84 years will be grouped into cognitively intact (*n* = 50, MoCA score ≥ 26) and cognitively impaired (*n* = 50, MoCA < 26). The CAVIRE performance-indices of cognitively healthy younger participants aged 35–64 years (*n* = 75) will serve as benchmark references. CAVIRE auto-computes the participant's performance-indices in 13 different segments. The tasks include domestic chores, memory, shopping, and social interactions. The proportion of participants who complete the entire VR assessment in each age group will be evaluated as feasibility indicators. The CAVIRE performance-indices will be compared across the different age groups. Feedback on the acceptability of the CAVIRE system will be collated and compared among the participants across the age groups.

**Significance:** CAVIRE is designed to assess the six domains of cognitive function using VR. The results of this feasibility study will provide insights for the implementation of the CAVIRE system as an alternative modality of cognitive assessment in the community.

## Introduction

### Background

Dementia refers to the decline and impairment of cognitive function to the extent that it interferes with one's daily activities. Based on the Diagnostic and Statistical Manual of Mental Disorders (DSM−5), cognitive function covers six domains: perceptual-motor function, executive function, complex attention, social cognition, learning and memory, and language (American Psychiatric Association, 2013). Dementia has been increasing in prevalence globally. According to the World Health Organization, an estimated 50 million people worldwide were diagnosed with dementia in 2019. This figure is projected to triple to 152 million by the year 2050 (World Health Organisation, 2019).

Dementia is also prevalent in Singapore, where ~82,000 of its residents had dementia in 2018; this figure is estimated to increase to 187,000 by 2050 (Alzheimer's Disease Association Singapore, 2019). This is particularly concerning due to Singapore's rapidly-aging population. In 2017, the proportion of those aged 60 years and older in the total population was 19.7%. This is predicted to reach 40% by the year 2050 (Malhotra et al., 2018). The expected increase in the number of persons with dementia is also linked with an increase in healthcare costs. In 2016, Singapore's healthcare expenditure for dementia was approximately SG$2.81 billion, and this is estimated to triple to SG$6.46 billion by 2050 (Woo et al., 2017). Hence, the number of older persons with dementia in Singapore and the associated healthcare expenditure are expected to increase significantly in the next 30 years.

Caregiver burden relating to dementia is another issue of concern, especially in context of the reducing family size in Singapore. Besides having to earn a living to support the household, the family breadwinner is expected to be the concurrent caregiver for their family member with dementia. Their care requires the caregivers to expend a large amount of time, energy, and finances, and to hire domestic helpers for assistance, leading to distress, and increased healthcare expenditure (Alzheimer's Disease Association Singapore, 2019).

In view of the significant healthcare costs and caregiver burden in managing dementia, it is important to identify dementia early. Early identification allows appropriate intervention to be initiated to retard the progression of dementia and more time to prepare the caregivers to co-manage the patients with healthcare professionals. A combination of diet modification, exercise, and cognitive training has been shown to delay the onset of dementia (Brasure et al., 2018).

In general, dementia presents as a spectrum of cognitive decline from the early stages of Mild Cognitive Impairment (MCI) to the late stages of Alzheimer's disease. For MCI, there is evidence of poor performance in one or more cognitive domains, but independence in performing daily activities is preserved ( Langa and Levine, 2014). Nonetheless, dementia is often diagnosed at a late stage, when significant impairment and dysfunction will have interfered with the patient's domestic, work and social activities, and relationships with their families and acquaintances (Petersen, 2016). Moreover, the full diagnostic workup of dementia can be costly, involving extensive medical examination, laboratory assessment, neurocognitive assessment, and neuroimaging (Langa and Levine, 2014). Assessing the six cognitive domains concurrently in a single setting can be challenging and requires significant time and effort by trained healthcare professionals. Therefore, an easily-executed and affordable modality of assessment is needed to complement the current modalities of assessment for MCI.

At present, the common methods of screening for cognitive impairment include questionnaire-based evaluations, such as the Montreal Cognitive Assessment (MoCA) and the Mini-Mental State Examination (MMSE) (Abd Razak et al., 2019). However, the questionnaires are limited in scope in identifying impairments across all six cognitive domains. For example, MMSE focuses on attention, orientation, memory, and language assessments, but is less sensitive in detecting executive function deficits, which are more evident in MCI (Kim et al., 2014). The assessment of activities of daily living (ADL) or instrumental activities of daily living (IADL), based on self-reporting by patients or observation by caregivers, can be subjective and may not be precise in assessing executive function. These questionnaires may not adequately cover a patient's day-to-day tasks in the actual home and community environment (Tarnanas et al., 2013; Gamito et al., 2017).

In recent years, virtual reality technology has been increasingly used in healthcare. Virtual reality (VR) is defined as an application by which participants engage in different senses to navigate and interact with three-dimensional computer-generated environments (Burdea and Coiffet, 2003). VR has applications in areas such as surgical simulation and training, rehabilitation, psychotherapy and medical education ( Pensieri and Pennacchini, 2014).

VR has also been used in screening for cognitive impairment. However, many existing VR tests rely on external controls such as a computer mouse or touchscreen to interact with a two-dimensional screen or augmented reality, instead of using a head-mounted device (HMD) with a three-dimensional environment (Bottiroli et al., 2017; Gamito et al., 2017; Zygouris et al., 2017; Chua et al., 2019). Without a three-dimensional environment, these VR systems are not be able to create a fully-immersive experience where the person has the sense of “being there,” thus being unable to simulate tasks that sufficiently mimic real life to assess a person's cognitive function (Strong, 2020). Moreover, these systems can only assess limited number of cognitive domains in one setting and lack language-domain assessment (Fernandez Montenegro and Argyriou, 2017; Seo et al., 2017; Lecouvey et al., 2019).

We have developed a novel fully-immersive VR system (known as CAVIRE or Cognitive Assessment by VIrtual REality). CAVIRE utilizes a head-mounted device, which allows the participants to perform virtual tasks in a three-dimensional environment with automated voice and visual instructions. These tasks are designed to assess their cognitive function in all the six domains.

### Aims

#### Primary Aim

The primary aim of this study is to evaluate the feasibility of using the CAVIRE system to assess cognitive function of participants aged from 35 to 84 years in a primary care clinic in Singapore.

Feasibility is determined by:
  - Calculating the proportion of participants who complete the entire CAVIRE assessment. We postulate that at least 90% of participants (23 out of 25 participants) in each considered age group (35–44 years; 45–54 years; 55–64 years; 65–74 years; and 75–84 years) are able to complete the entire CAVIRE assessment. Participants who drop out from the study due to headache, nausea, or giddiness during the assessment are considered non-completers.
  - Comparing the time taken by the participants to complete the CAVIRE assessment vs. the time taken to complete the questionnaire-based assessments [Montreal Cognitive Assessment (MoCA), Abbreviated Mental Test (AMT), Mini-Mental State Examination (MMSE)]. We hypothesize that the time taken to complete the CAVIRE assessment will be equal to or less than that of the questionnaire-based assessments.

#### Secondary Aims

The secondary aims of this study include:

1. To determine the CAVIRE performance-indices (completion time and scores) of the participants who undergo the assessment. This will be evaluated by:
  - Comparing the CAVIRE performance-indices of cognitively healthy participants, between those aged 35–64 years (younger age group) with those aged 65–84 years (older age group). We postulate that participants in the younger age group will have better performance-indices compared to those in the older age group (Harada et al., 2013).
  - Comparing the CAVIRE performance-indices of cognitively healthy participants vs. cognitively impaired participants in the older age group (65–84 years). Prior to the CAVIRE assessment, we will be adopting a MoCA cut-off score of 26 to categorize the cognitive status of the older participants, based on local data in Singapore. A MoCA score of 26 or more suggests a cognitively healthy status, while a score of less than 26 suggests a cognitively impaired status (Ng et al., 2013). We hypothesize that the CAVIRE performance-indices of the cognitively healthy participants will be higher than the cognitively impaired participants.

2. To evaluate the acceptability of participants toward the use of CAVIRE as a modality for cognitive assessment. This will be evaluated by:
  - Assessing the participants' response through a feedback form upon completion of the CAVIRE assessment. This will also include the participants who are not able to complete the CAVIRE assessment due to headache, nausea or giddiness. We hypothesize that there will be an overall positive response from the participants toward the use of CAVIRE as a cognitive assessment tool in the primary care clinic.

## Methods

### Development of the CAVIRE System

The CAVIRE system is developed by a multidisciplinary team comprising family physicians, academics with special interest in geriatrics, engineers from a local technical institution and a VR specialized company. It comprises of: (A) a tutorial session at the start, (B) 13 different segments of cognitive assessment, and (C) an automated scoring system. The total given time to complete the tutorial and assessment is <15 min.

#### Tutorial Session

Participants will be guided on how to interact with the VR environment by using head movements and hand movements respectively.

#### 13 Segments of Cognitive Assessment

Each of the 13 segments consists of tasks that assess specific domains of cognitive function. These tasks are common day-to-day activities. They can be done via hand gestures and head movements which are detected by motion sensors, or via speech which is detected by a voice recognition device. The participants follow automated voice and visual instructions to complete the respective tasks. The medium of instruction is English.

The participant will undertake VR tasks across 13 different segments:

1. Brushing and rinsing teeth
2. Preparing peanut butter bread for breakfast
3. Identifying pictures of important persons in the newspaper
4. Watching television, while listening to the weather forecast regarding impending rain on the radio
5. Naming the fruits in a shopping list and remembering the fruits
6. Choosing the appropriate clothing to go for grocery shopping
7. Remembering to pick up the umbrella, before opening and locking the door
8. Taking the lift to level 1 in an apartment block by pressing the correct buttons
9. Looking to the left and right, and waiting for green pedestrian light, before crossing the street
10. Remembering and choosing the stipulated stall, i.e., the one which sells fruits
11. Picking the correct fruits based on recall from the shopping list
12. Calculating and paying the correct sum of money for all the fruits
13. Selecting the appropriate emotion, with regards to scenes of a birthday party and car accident respectively.

**Figure 1:**
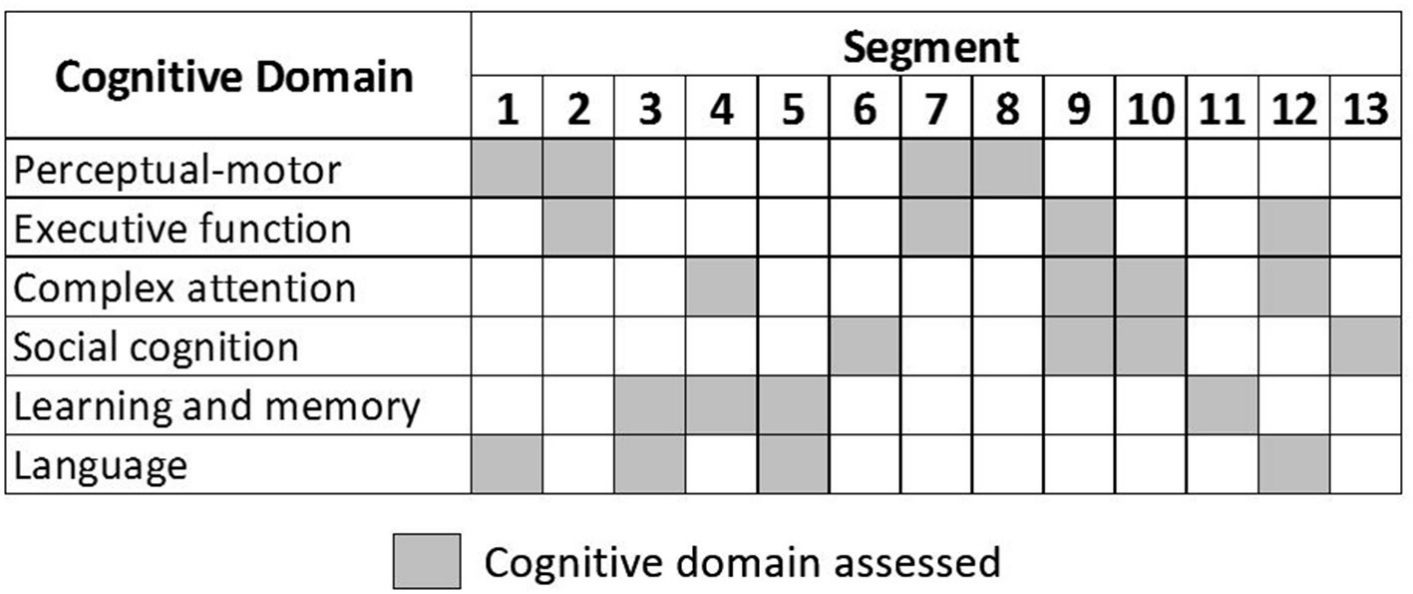
Cognitive domains assessment in each segment.

The segments assess all six cognitive domains, as depicted in Figure 1. Some segments contain tasks that assess more than one cognitive domain. To achieve a balanced assessment, each cognitive domain is assessed across four different segments. The system was pilot-tested internally with young and older volunteers over three rounds. The volunteers provided feedback regarding: the three-dimensional environment, visual and voice instructions, interaction with objects to perform the tasks, and overall user-friendliness of the CAVIRE system. The feedback resulted in modifications for improving the CAVIRE system. For example, optimizing the sensitivity of grabbing and releasing objects, and removing unnecessary animations that may cause visual discomfort.

#### Automated Scoring System

An automated scoring system to assess each task performance is embedded in the CAVIRE system. Upon completion of all the 13 segments, the scores will be computed automatically and presented to the investigator as scores in each segment and for the entire assessment. The scoring matrix is based on (a) the proportion of tasks performed correctly; (b) number of attempts needed to complete each task; and (c) completion of tasks within the given time. The scoring algorithm for all 13 segments of the VR assessment is shown in “APPENDIX 1—Scoring algorithm of the VR assessment”.

The scoring for number of attempts needed varies for each Segment. For example, in segment 3 the participant is only allowed one attempt to identify each image of the important persons in the newspaper; in segment 12 the participant is allowed more than one attempt to calculate the total price of the five fruits. Please refer to APPENDIX 1 for more details.

#### Recording of time Taken to Complete the CAVIRE Assessment

There is a maximum time limit for the participant to complete the tasks in each segment. Some participants may take a shorter time to complete a particular segment, while others may take a longer time. That time taken for each segment will be recorded by the system.

However, if the participant is unable to complete a particular segment when the maximum time limit has reached, the system will automatically proceed to the next segment. In this case, the maximum time limit will be recorded as the time taken for that particular segment.

At the end of the assessment, the system will sum up the time taken to complete all segments. That sum will be compared with the time taken to complete the questionnaire-based assessments.

#### Future Development of the CAVIRE System

Currently we are in the first phase of the development of the CAVIRE system, where we are assessing its feasibility. If the feasibility results are promising, we will be performing content validation in the next phase, where additional funding and resources would be required.

### Study Design

This is a pilot study at a single site. It will evaluate the feasibility of the CAVIRE system, which is a novel fully-immersive VR system to assess cognitive function. Feasibility is evaluated by the proportion of participants who complete the entire CAVIRE VR assessment in each age group. The study has been initiated in October 2020 and is estimated to be completed by August 2021. This protocol adheres to recommendations from the “The Standard Protocol Items: Recommendations for Interventional Trials (SPIRIT)” (Chan et al., 2013).

### Study Site

The study will be conducted at a public primary care clinic (polyclinic) in Outram, located in southern Singapore. This polyclinic provides subsidized primary healthcare services to approximately 18,960 residents of varying ethnicity in the estate, of which 24.7% were aged 65 years and above in 2019 (Department of Statistics, 2019). The in-house GeRiAtric serviCE (GRACE clinic) is a sub-specialized memory clinic to provide longer consultation time to assess older participants with suspected cognitive impairment.

### Participants

The target participants are those who attend the polyclinic for medical consultation, in both the general clinic and the GRACE clinic. Visitors and accompanying persons of patients at the polyclinic will also be recruited if they satisfy the eligibility criteria.

#### Inclusion Criteria

1. Age between 35 and 84 years old;
2. Understand English (the medium of instruction in CAVIRE);
3. Willing to complete the questionnaires and the CAVIRE VR assessment.

#### Exclusion Criteria

1. Pre-existing diagnosis of cognitive impairment or dementia as documented in their electronic medical record;
2. Any disability which renders the potential participant incapable of providing written informed consent;
3. Having neurological deficits that may affect vision, hearing, speech or motor skills;
4. Having previous history of conditions (e.g., motion sickness, epilepsy) that may be triggered as a result of exposure to virtual reality.

### Sample Size

The study will recruit a total of 175 participants. 25 participants with MoCA score ≥26 will be enrolled in each of the following 10-year age groups to determine the VR performance: (1) 35–44 years; (2) 45–54 years; (3) 55–64 years; (4) 65–74 years; (5) 75–84 years. In addition, 25 participants with MoCA score <26 (cognitively impaired) will be recruited in the (1) 65–74 years; (2) 75–84 year age groups.

A sample size of 12–30 individuals is recommended for feasibility studies (Billingham et al., 2013). In this feasibility study, a sample size of 25 participants for each considered age group will be used. If at least 90% of participants (23 out of 25 participants) in each age group are able to complete the VR assessment, we can be 95% confident that at least 77% of future participants, in that age group, who undertake the VR assessment will be able to complete the assessment.

### Recruitment

The investigators will screen and identify potential participants at the waiting area of the general clinics, and also via referral from the GRACE clinic. The investigators will introduce the research study to the potential participants, and also assess the inclusion and exclusion criteria. The participants will then undergo the Montreal Cognitive Assessment (MoCA).

For older participants aged 65–84 years old, those with MoCA score >26 will be considered cognitively healthy, while those with MoCA score <26 will be considered cognitively impaired. A +1 point correction will be done for older adults with ≤ 10 years of education (Ng et al., 2013). For both cognitively-healthy and cognitively-impaired groups, 25 participants will be recruited for each of the age groups: 65–74 years, 75–84 years. Once the target of 25 participants is attained, recruitment will end for that particular age group. If the participant has MoCA score <26, the participant will be referred for further clinical assessment at the end of the study.

For younger participants aged 35–64 years old, most of them are expected to be cognitively healthy, hence only those with MoCA score >26 will be recruited. 25 participants will be recruited for each of the age groups: 35–44 years, 45–54 years, and 55–64 years. Once the target of 25 participants is achieved, recruitment will end. However, if the participant happens to have MoCA score <26, it will be considered an incidental finding of cognitive impairment, subsequently the participant will be referred for further clinical assessment. The participant will not be recruited for the study.

Upon completion of the MoCA assessment, the investigator will provide the participants with an information document regarding the study. The participants will be given time to understand and consider their participation in the study, and for the investigators to clarify their queries. Once their concerns and queries are addressed, written consent will be taken by the investigator in the presence of a witness. The investigator will also seek consent from the participants to access the electronic medical records to verify their eligibility criteria. Next, the participants will proceed with the pre-VR questionnaires, followed by the CAVIRE assessment, and finally the post-VR feedback form to complete the study.

**Figure 2:**
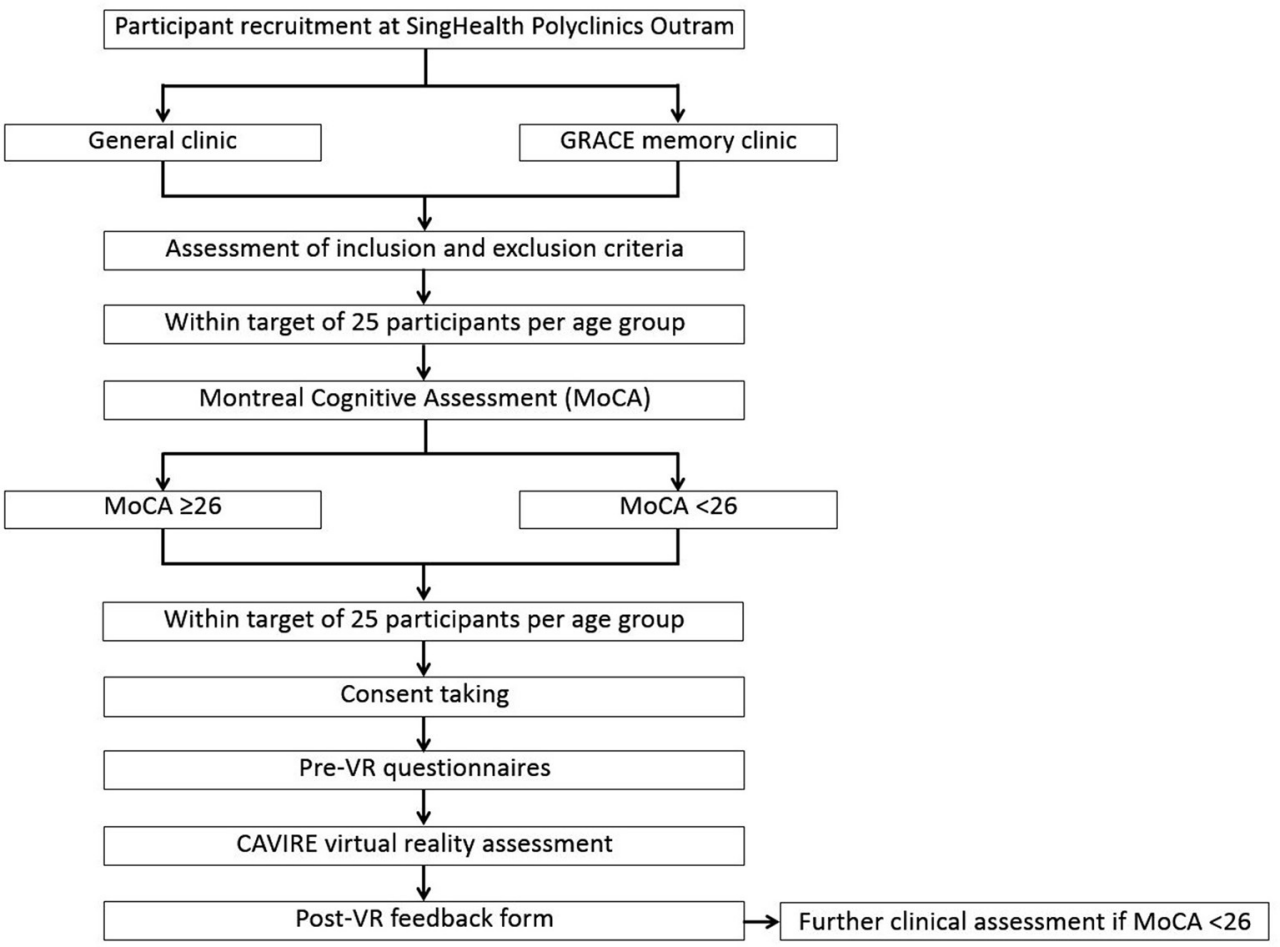
Flowchart of the recruitment process for participants aged 65–84 years.

**Figure 3:**
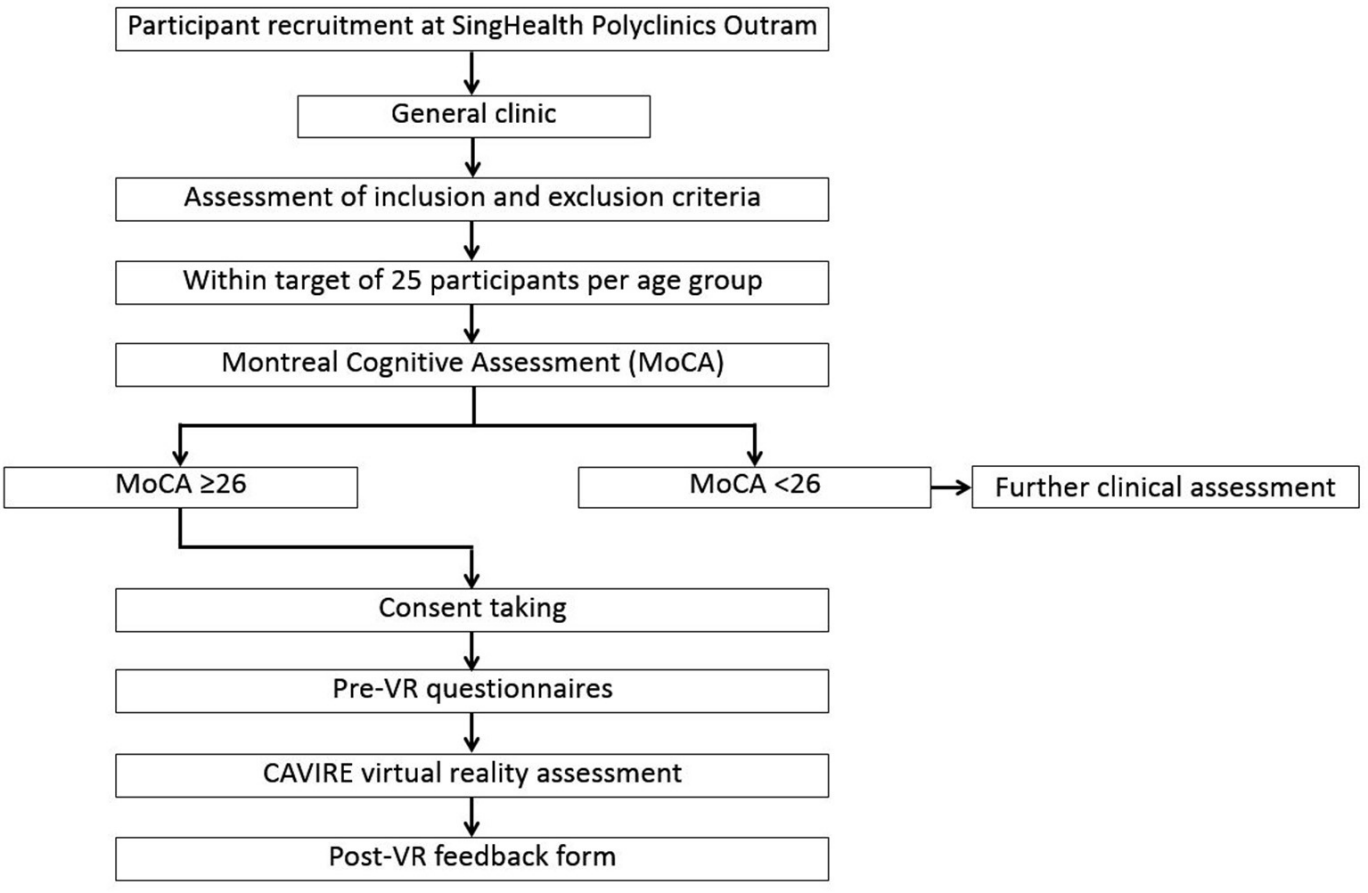
Flowchart of the recruitment process for participants aged 35–64 years.

A summary of the recruitment process for participants aged 65–84 years is depicted in Figure 2, while the recruitment process for those aged 35–64 years is shown in Figure 3.

### Pre-VR Questionnaires

The investigator will administer questionnaires to collect demographic data and assess cognitive status based on the validated cognitive and functional status assessment tools prior to the VR assessment.

- Demographic characteristics (age, gender, ethnicity, and number of years of education)
- Cognitive assessments [Abbreviated Mental Test (AMT), Mini-Mental State Examination (MMSE)]
- Functional status assessments (Barthel Index—Basic Activities of Daily Living (BADLs) (Mahoney and Barthel, 1965), Lawton Instrumental Activities of Daily Living (IADLs) Scale (Graf, 2008).

For each of the MoCA, AMT, and MMSE questionnaires, the investigator will indicate the “start” time and “end” time respectively. The time taken to complete these 3 questionnaires will then be calculated.

### Procedure in VR Assessment

Participants will be briefed regarding the VR procedure. After introducing the equipment, the participants will be seated on a chair in a fixed position. They will put on the VR head-mounted device with the help of the investigator. They will then be guided through the CAVIRE-based tutorial to familiarize themselves with the VR 3-D environment. The participant will proceed to complete the 13 segments. The time taken and performance scores will be automatically calculated by the system.

After the VR assessment, the participants will provide feedback by filling in a questionnaire on their experience in using the VR system. This questionnaire is adapted from the validated Spatial Presence Experience Scale (SPES) (Hartmann et al., 2016). Refer to “APPENDIX 2—Feedback form after VR assessment.” The questions will collect data on their (1) level of comfort throughout the VR test; (2) perception of completing daily living tasks in the virtual environment; and (3) level of motivation and interest on the use of virtual technology in general practice. For each question, the answers will be rated on a Likert scale (from 1 to 5, corresponding from “strongly disagree” to “strongly agree”).

### Outcome Measures

#### Primary Outcome

The feasibility outcome is determined by the proportion of participants who complete the entire CAVIRE assessment across the different age groups. This study will be considered a success if at least 90% of participants (23 out of 25 participants) in each age group are able to complete the CAVIRE assessment. Participants who drop out due to headache, nausea or giddiness during the assessment are considered non-completers.

Feasibility is also measured by comparing the time taken to complete the CAVIRE assessment with the time taken to complete the questionnaire-based assessments (MoCA, AMT, and MMSE) in the different age groups, hypothesizing the time taken to complete the CAVIRE assessment to be equal to or less than that of the questionnaire-based assessments. Paired *t*-test, or its non-parametric equivalent, the Wilcoxon signed-rank test, will be used as appropriate for comparing the time taken for the two assessments overall and in each age group. However, if the time taken to complete the CAVIRE assessment is significantly longer compared to that of the questionnaire-based assessments, then the existing CAVIRE would not be appropriate as a screening tool for cognitive impairment. Further design and modifications to the individual segments and tasks of the CAVIRE system would need to be implemented.

#### Secondary Outcomes

1. Comparison of the CAVIRE performance-indices across age groups and cognitively distinct groups to assess its discriminatory functions. This will be assessed by the performance-indices between the younger age groups and the older age groups among those with MoCA scores ≥26; and also between the cognitively healthy and the cognitively impaired participants in the older age groups based on the MoCA score cut-off of 26. A positive outcome is attained if participants in the younger age group achieve better performance-indices compared to those in the older age group; and if cognitively healthy participants achieve better performance-indices compared to cognitively impaired participants.
2. Acceptability of participants toward the use of CAVIRE as a modality for cognitive assessment to assess its potential implementation in primary care. This will be evaluated by a questionnaire survey. The scope of the questions involves (a) level of comfort throughout the VR test; (b) perception of completing daily living tasks in the virtual environment; and (c) level of motivation and interest on the use of virtual technology in general practice. The answers will be rated on a Likert scale and a total score will be calculated. A rating of 80% and above of the maximal total scores among the participants will be considered a positive outcome.

### Data Management and Monitoring

The data from the questionnaires will be transcribed into the Redcap database, and will be audited by a data management officer for data entry errors. The data from the CAVIRE system will be exported to the same database and merged with the audited questionnaire data. To protect the participants' confidentiality, their identifiers will be stored separately from the research data. All the study data will be stored in the password-protected database, which is accessible only by the investigators. The questionnaires will be kept in locked cabinets at the study site and will be accessible for audit by the monitoring authorities.

### Adverse Events Monitoring

In the event that the participant complains of any headache, nausea or giddiness during the VR assessment, the assessment will be terminated. The participant will be instructed to take off the VR head-mounted device and rest to recover from the symptoms. The participant will not be allowed to continue with the VR assessment and will be considered as a dropout. If the symptoms persist despite the rest, the participant will be attended by a polyclinic physician for further management. The number of participants who fail to complete the study and the reasons will be documented and reported.

### Statistical Methods

Summary statistics will be calculated for the proportion of participants who complete the entire VR assessment in each considered age group, the demographic characteristics, the completion time and scores for the questionnaires (MoCA, AMT, and MMSE), the completion time and scores for the VR tasks, and the scores for the feedback form respectively.

The comparison of the time taken to complete the VR assessment with the time taken to complete the cognitive assessments (MoCA, AMT, and MMSE) will be assessed using the paired *t*-test, in consideration that the data for time taken is continuous and is assumed to follow a normal distribution. However, in the event that the data is not normally distributed, the non-parametric Wilcoxon signed-rank test would be used instead. The tenability of the normality assumption will be assessed for each response variable and an appropriate analysis approach employed, either parametric or non-parametric.

The VR performance-indices (completion time and scores) will be analyzed at the level of each of the 13 segments. The VR performance-indices will be compared among the cognitively healthy participants, between those aged 35–64 years (younger age group) with those aged 65–84 years (older age group). The performance-indices will also be compared among the older age group (65–84 years), between those who are cognitively healthy with those who are cognitively impaired. These comparisons will be done via two-sample parametric and non-parametric statistical methods as appropriate. Multivariable analysis of covariance (ANCOVA) will also performed to take into account any possible demographic confounders such as gender, race, and number of years of education.

For each of the statistical tests, a *P*-value of <0.5 will be considered statistically significant (two-sided). The statistical analyses will be performed using the SAS University Edition software.

## Discussion

### Overview

CAVIRE is a fully-immersive and automated VR system designed to assess the six domains of cognitive function. The automated audio–visual instructions and scoring matrix embedded in the VR system standardize the assessment procedure and avoid inter-rater variations. Differential performance-indices between the cognitively healthy and the cognitively impaired participants in the older age groups will suggest its potential as an alternative modality of cognitive assessment. The data will identify potential design deficiencies in each segment of the VR assessment and to allow enhancement and modification to improve its content, design and delivery. Subsequently, further studies will be done to examine the implementation and scalability of the CAVIRE system in the healthcare setting.

### Potential Limitations of the Study

Younger participants are postulated to perform better in the VR assessment due to their wider exposure to gaming and prior experience with VR (Davison et al., 2017). The tutorial session embedded prior to the start of CAVIRE assessment attempts to mitigate such advantages. The participants can repeat the tutorial to gain confidence in their performance, so that they begin the VR assessment at a comparable level of confidence and competency.

Although MoCA has its limitations in assessing cognitive function, it is commonly used to differentiate the cognitive status of the participants in clinical practice. Alternative tools are also limited by practicality and validity issues in routine usage.

Currently, the CAVIRE system uses the English language as the medium of instruction for cognitive assessment. Future development of the VR software will incorporate other local languages such as Chinese and Malay to deliver the instructions.

We recognize the potential of selection bias in our study. Due to the nature of this feasibility study, it is not possible to perform random sampling during recruitment. Nevertheless, if the results of this feasibility study are favorable, random sampling will be applied in future studies.

## Summary

In summary, CAVIRE has the potential to become a screening tool for cognitive impairment. By sharing our protocol with the wider research community, we would like to contribute to the state of development of virtual reality technology in assessing cognitive function. The results of this study would be able to advance the frontier of applying medical technology to explore new modalities of cognitive assessment in clinical practice.

## Ethics Statement

The studies involving human participants were reviewed and approved by SingHealth Centralized Institutional Review Board of Singapore. The patients/participants provided their written informed consent to participate in this study.

## Author Contributions

JEL, WTW, JHMQ, and NCT designed the VR performance tasks, score matrix, and the study protocol. RM reviewed the face validity of the 13 segments for cognitive assessment. TAT and SHL collaborated with FXMedia Internet Pte. Ltd., the industrial collaborator to develop the VR system. WTW and NCT secured the funding. For this protocol manuscript, JEL and NCT drafted the manuscript. The other investigators reviewed and critiqued. The draft is revised, finalized, and approved by all investigators before submitting the manuscript to the journal. All authors contributed to the article and approved the submitted version.

## Conflict of Interest

The authors declare that the research was conducted in the absence of any commercial or financial relationships that could be construed as a potential conflict of interest.
