# Supplementary material for: A Fully-Immersive and Automated Virtual Reality System to Assess the Six Domains of Cognition: Protocol for a Feasibility Study
Source: Front Aging Neurosci. 2021 Jan 7;12:604670. doi: 10.3389/fnagi.2020.604670 (PMC7817896; doi:10.3389/fnagi.2020.604670)
Supplement: Supplementary file 1 [file Data_Sheet_1.docx]

**APPENDIX 1 – Scoring algorithm of the VR assessment**

| **Segment** | **Task** | **Score** | | | | | **Remarks** | **Cognitive**  **Domain(s)**  **assessed** |
| --- | --- | --- | --- | --- | --- | --- | --- | --- |
|  |  | **0** | **25** | **50** | **75** | **100** |  |  |
| 1 | Following step-by-step instructions:  1) Squeeze toothpaste on toothbrush 2) Brush teeth  3) Rinse mouth | No attempt | Attempts, but unable to complete any Tasks | Complete 1 Task | Complete 2 Tasks | Complete all 3 Tasks | NIL | Perceptual motor  Language |
| 2 | Preparing peanut butter bread without specific instructions:  1) Open peanut butter jar  2) Take peanut butter using knife  3) Spread peanut butter on bread | No attempt | Attempts, but unable to complete any Tasks | Complete 1 Task | Complete 2 Tasks | Complete all 3 Tasks | NIL | Perceptual-motor  Executive function |
| 3 | Identify 3 images of important persons in the newspaper: Lee Kuan Yew, Halimah Yacob, Goh Chok Tong | No attempt | Attempts, but unable to identify any images correctly | Identify 1 image correctly | Identify 2 images correctly | Identify all 3 images correctly | NIL | Learning & memory  Language |
| 4 | 1) Remember to take umbrella before leaving the house later  >> Television acts as a distractor  >> Radio gives weather forecast of rain | NIL | NIL | NIL | NIL | NIL | This Task will be scored in Segment 7(b) | Complex attention  Learning & memory |
| 5 | 1) Name the 5 fruits by reading out aloud: Apple, Banana, Watermelon, Mango, Durian  2) Remember the 5 fruits | No attempt | Attempts, but unable to name any fruits correctly | Name 1-2 fruits correctly | Name 3-4 fruits correctly | Name 5 fruits correctly | Task #2 will be scored in Segment 11 | Learning & memory  Language |
| 6 | 1) Choose appropriate clothing (female/male) to go out for shopping | No attempt | Attempts, but unable to choose the correct clothing | Choose the correct clothing in 2 attempts or more | Choose the correct clothing in 1 attempt, with a time of 15 seconds or more | Choose the correct clothing in 1 attempt, with a time of less than 15 seconds | NIL | Social cognition |
| 7(a) | 1) Open the door  2) Select the correct item to lock the door  3) Lock the door | No attempt | Attempts, but unable to complete any Tasks | Complete 1 Task | Complete 2 Tasks | Complete all 3 Tasks | NIL | Perceptual-motor  Executive function |
| 7(b) | Remember to take umbrella before leaving the house  >> Hint given at two time points:  (i) before locking the door  (ii) after locking the door | Does not remember to take the umbrella at all | Remember to take the umbrella after locking the door (hint is given for the second time) | Remember to take the umbrella before locking the door (hint is given for the first time) | Remember to take the umbrella after opening the door (before any hints are given) | Remember to take the umbrella before opening the door (before any hints are given) | Continued from Segment 4 | Continued from Segment 4 |
| 8 | 1) Press button to go down (outside lift)  2) Press button for Level 1 (inside lift) | No attempt | Attempts, but unable to complete any Tasks | Complete both Tasks in 2 attempts or more respectively | Complete 1 Task in 1 attempt &  Complete the other Task in 2 attempts or more | Complete Task #1 in 1 attempt & Complete Task #2 in 1 attempt | NIL | Perceptual-motor |
| 9 | 1) Press “Start” to cross after traffic light turns green  2) Looks to the left before crossing  3) Looks to the right before crossing | No attempt | Attempts, but unable to complete any Tasks | Complete 1 Task | Complete 2 Tasks | Complete all 3 Tasks | NIL | Executive function Complex attention  Social cognition |
| 10 | Choose the correct store (fruits) from a row of stores: clothes, electrical appliances, vegetables, fruits | No attempt | Attempts, but unable to choose the correct store | Choose the correct store in 2 attempts or more | Choose the correct store in 1 attempt, with a total time of 15 seconds or more | Choose the correct store in 1 attempt, with a total time of less than 15 seconds | NIL | Complex attention  Social cognition |
| 11 | Choose the 5 fruits based on the previous shopping list: Apple, Banana, Watermelon, Mango, Durian | No attempt | Attempts, but unable to choose any fruits correctly | Choose 3 fruits or less correctly | Choose 4 fruits correctly | Choose 5 fruits correctly | Continued from Segment 5 | Learning & memory |
| 12 | Following step-by-step instructions:  1) Calculate total price of the 5 fruits  2) Pay exact amount of money | No attempt | Attempts, but unable to complete any Tasks | Complete both Tasks in 2 attempts or more respectively | Complete 1 Task in 1 attempt, & Complete the other Task in 2 attempts or more | Complete Task #1 in 1 attempt, & Complete Task #2 in 1 attempt | NIL | Executive function Complex attention  Language |
| 13 | Choose the correct emotion with regards to the scene:  1) Birthday party  2) Car accident | No attempt | Attempts, but unable to complete any Tasks | Complete both Tasks in 2 attempts or more respectively | Complete 1 Task in 1 attempt, &  Complete the other Task in 2 attempts or more | Complete Task #1 in 1 attempt, & Complete Task #2 in 1 attempt | NIL | Social cognition |

**APPENDIX 2 – Feedback form after VR assessment**

| **Participant Feedback Form**  **Thank you for participating in this virtual reality (VR) test to assess for cognitive function.**  **Please rate on a scale of 1 to 5 for the following questions.**  **Tick (🗸) the box most relevant to you.** |
| --- |

| **Date of VR Assessment** | **______________ (dd/mm/yyyy)** |
| --- | --- |

|  | Strongly Disagree  **1** | Disagree  **2** | Undecided  **3** | Agree  **4** | Strongly Agree  **5** |
| --- | --- | --- | --- | --- | --- |
| **Level of comfort in using Virtual Reality** | | | | | |
| 1. The virtual reality (VR) system was easy to use. |  |  |  |  |  |
| 1. The amount of time I spent on the VR test is acceptable to me. |  |  |  |  |  |
| 1. During the VR test, I did not experience any symptoms such as: nausea, headache or giddiness. |  |  |  |  |  |
| **Level of similarity of Virtual Reality compared to the real world** | | | | | |
| 1. It seemed that I was actually there in a new environment. |  |  |  |  |  |
| 1. The environment seemed similar to the real world. |  |  |  |  |  |
| 1. I was able to interact with the objects around me to perform the tasks. |  |  |  |  |  |
| **Level of interest towards Virtual Reality** | | | | | |
| 1. The use of VR helps to make the experience in the clinic more interactive. |  |  |  |  |  |
| 1. The use of VR to help diagnose medical condition appeals to me. |  |  |  |  |  |
| 1. In the future, I would like to see more VR applications being used in the clinic. |  |  |  |  |  |
| **Overall experience** |  |  |  |  |  |
| 1. Overall, I enjoyed the VR experience in the clinic. |  |  |  |  |  |
